# Cooperative learning in the first year of undergraduate medical education

**DOI:** 10.1186/1477-7819-5-136

**Published:** 2007-11-28

**Authors:** Rani Kanthan, Sheryl Mills

**Affiliations:** 1 Department of Pathology and Laboratory Medicine, College of Medicine, University of Saskatchewan, Saskatoon, Saskatchewan, Canada; 2 Department of Educational Administration, College of Education, University of Saskatchewan, Saskatoon, Saskatchewan, Canada

## Abstract

**Background:**

Despite extensive research data indicating that cooperative learning promotes higher achievement, the creation of positive relationships, and greater psychological health for students at all levels in their education, cooperative learning as a teaching strategy is still underutilized in undergraduate medical education.

**Methods:**

A cooperative learning task was introduced as part of the mandatory first Year undergraduate Pathology course. The task was to create an 8.5" × 11" poster summary of pre-assigned content in self-chosen groups of four or five students. On the designated "Poster Day," the posters were displayed and evaluated by the students using a group product evaluation. Students also completed an individual group process reflection survey. An objective evaluation of their understanding was gauged at the midterm examination by specific content-related questions.

**Results:**

Majority (91–96%) of students judged the group products to be relevant, effective, easy-to-understand, and clearly communicated. The majority of the students (90–100%) agreed that their group process skills of time management, task collaboration, decision-making and task execution were effective in completing this exercise. This activity created a dynamic learning environment as was reflected in the students' positive, professional discussion, and evaluation of their posters. The content-related questions on the midterm examination were answered correctly by 70–92% of the students. This was a mutually enriching experience for the instructor and students.

**Conclusion:**

These findings demonstrate that cooperative learning as a teaching strategy can be effectively incorporated to address both content *and *interpersonal skill development in the early years of undergraduate medical education.

## Background

The current health care system promotes patient-centered medicine through inter-professional collegiality and teamwork [1-3]. Undergraduate medical education is traditionally structured largely around faculty authority and lectureship which encourages individualistic competitive environments. Extensive research data indicates that cooperative learning promotes higher achievement and greater psychological health for students at all levels in their education, in addition to creating positive interpersonal relationships [4] – a fundamental component of effective teamwork. Group work, in its various forms such as collaborative learning, teams, small groups, task teams, problem-based learning groups, case-based groups and others, is not unusual in medical practice and in the final years of clinical training [5].

However, five key elements distinguish Cooperative Learning (CL) from other forms of group work [4,6,7]. These are:

(1) *face-to-face interaction *amongst students and their peers;

(2) *individual accountability *promoting personal responsibility through individual exams or self and peer assessment;

(3) *group processing *wherein group members reflect on the group skill process and make decisions about what to continue and what to change;

(4) *positive interdependence *created through establishing group goals, group tasks, team roles, learning goals, rewards, or shared resources; and

(5) *interpersonal skills *such as decision-making, leadership, trust-building, communication, conflict management, perseverance, and seeking to understand are specifically taught and practiced in this setting.

These distinguishing features of cooperative learning promote student engagement by providing students with opportunities for discussion, problem-solving, consensus building, team building, power sharing, and trust building[7] leading to enthusiasm and a sense of mutuality. The group skills that students practice in cooperative learning activities are transferable to problem-based learning, self-directed learning, and experiential learning which are being increasingly used in undergraduate and post-graduate medical education [5,8,9].

Cooperative learning is a pedagogical teaching strategy designed to promote productive and mutual learning amongst a group of students and "to maximize the learning of all individuals in the group [10]." Students interact in purposively structured heterogeneous groups to support the learning of themselves and others in the same group [11]. Cooperative learning is student-centered, and an alternative to traditional curriculum-driven teacher-centered education. Learners work together in small groups to develop their own answers through interaction and reaching consensus, and *not *necessarily towards a pre-determined *right *answer.

Despite the overwhelming positive published efficacy of this methodology for learning to work in teams [12] and in the transfer of knowledge [13,14] cooperative learning as a teaching and learning strategy is still underutilized in higher education such as colleges and universities including undergraduate medical education. Some literature exists regarding the role of cooperative learning in the training of junior hospital doctors [15] and in an elective self-help group class in medical studies [16]. However, there is a paucity of published articles involving the use of cooperative learning in the early years of medical education. Given the overwhelming published benefits of cooperative learning at other levels of education the time was right to explore the benefits of cooperative learning in the first year Pathology course in the revised undergraduate medical curriculum at the University of Saskatchewan.

## Aim

The overall objectives for introducing cooperative learning in this Pathology course were to (a) encourage student ownership of learning, (b) shift the learning environment from an individualistic competitive system to a cooperative non-competitive atmosphere, (c) assess the feasibility of incorporating this strategy in the limited contact hours of the content-laden undergraduate medical curriculum, and (d) gauge students' responses to working in groups at this early embryonic phase of their medical careers.

The specific learning objectives for the students, on the other hand, were primarily two-fold: (i) to effectively synthesize the designated content, and (ii) to practice skills of decision-making, time management, consensus-building, trust-building, and group collaboration. As these were first year students of two colleges – medical and dental – we felt it was important for students to get to know each other by working together while learning thereby valuing, honoring, and respecting professional collegial behaviors both in and out of class time.

## Methods

### The context

Ninety first Year medical and dental students enrolled in the compulsory undergraduate Pathology course at the University of Saskatchewan participated in this study approved by the institution's ethical review committee. The aim of this course was to introduce the students to the general pathological principles and conditions common to the underlying systemic afflictions of the human body as applicable to the real life practices of medicine and dentistry. Students were made aware that they would be actively participating in a variety of instructional experiences that promote and help interweave the threads of understanding which link the pathology of diseases through multiple disciplines relevant to their careers. During the first week of the course, students were introduced to the philosophy of active learning to encourage student engagement with ownership of learning. They were made aware of course expectations regarding the incorporation of a wide variety of instructional methods which included a) formative evaluation of designated reading assignments, in-class discussions, debates, jig-sawing course material, and b) summative evaluation using multiple-choice and short answer questions on the midterm exam.

### The co-operative learning task

The distinguishing feature of cooperative learning is the attainment of two distinct goals: (a) the group creates a viable group product, and (b) that the groups' process maintains the integrity of the interpersonal relationships. In addition, there are five elements that distinguish Cooperative Learning (CL) from other forms of small group learning:

(1) face-to-face interaction (CL1),

(2) individual accountability (CL2),

(3) group processing (CL3),

(4) positive interdependence (CL4), and

(5) interpersonal skill development (CL5).

The cooperative learning task in this study was to create an 8.5" × 11" poster that effectively synthesized the subject content. This was the group product. The group process goals included the interpersonal skills of: effective time management, task collaboration/cooperation, effectiveness of decision-making strategies, and the valued apportioning of individual member's contributions. As indicated above, cooperative learning tasks have five distinguishing features. The relationship of this cooperative learning task is linked with the five distinguishing elements of cooperative learning in Table 1 and is further explained below.

**Table 1:** Relationship of Task to the Five Elements of Cooperative Learning. This figure categorizes the five elements of cooperative learning in relation to this task in the undergraduate first year Pathology course.

**Task**	**CL1**	**CL2**	**CL3**	**CL4**	**CL5**
Group Product (poster)	***X***	***X***	***X***	***X***	
Group selection	***X***			***X***	***X***
Signing poster		***X***			
Group Process Reflection			***X***		***X***
Exam questions		***X***			

CL1. face-to-face interaction

CL 2. individual accountability

CL 3. group processing

CL 4. positive interdependence, and

CL 5. interpersonal skill development.

#### Logistics of the cooperative learning task

1. Students were first informed of this task three weeks prior to the due date of the group product. They were responsible for learning and summarizing the chemical mediators of inflammation based on material from the recommended text book. The teams were given the task of creating an 8.5" × 11 inch poster synthesis of this material. (Table 1 – CL 1, 2, 3, 4)

2. Students were asked to choose their own groups of four or five (Table 1 – CL 1, 4, 5). Students formed groups based on who they felt they could best work with and with whom they could easily arrange 'out-of-class' meeting times.

3. Each group member acknowledged ownership by signing their poster (Table 1 – CL 2).

4. On the designated "Poster Day,"

a) the posters were displayed in the hallway.

b) each group reflected on and evaluated another group's poster (product) using the criteria identified in the pre-designed form (Appendix 1).

c) At the end of the session, students completed an individual group process reflection (Appendix 2). (Table 1 – CL 3, 5)

5. An objective evaluation of their understanding was gauged at the midterm examination by specific content-related questions (Table 1 – CL 2).

### Measurement tools

Two surveys were designed to measure the group product (Appendix 1) and the group process (Appendix 2).

• The group product was assessed with a subjective survey (Group Product Evaluation) based on modified 1 (yes) – 5 (no) Likert scale of effective synthesis, representation of relevant information, clear communication, and ease of understanding (Appendix 1).

• The group process was assessed with a subjective survey (Group Process Reflection) based on a modified 1 (yes) – 5 (no) Likert scale (of yes to no) of effective team time management, cooperative task execution, decision-making, team member contribution, and task strategy. This survey (Appendix 2) also invited students to share additional comments and observations including positive and negative feedback.

In addition, on the midterm examination, 9 of 67 multiple choice and 2 of 12 short answer questions specifically targeted the content synthesized on the poster.

### Data analysis

The *Group Process Reflection *was analyzed in a summative semi-quantitative fashion related to the criterion-related questions. The students' comments and observations were subjected to qualitative assessment by thematic categorical analysis. The responses to the midterm examination questions were analyzed quantitatively based on a percentage scale. We recognize this study was limited in two ways: (a) feedback was self-reporting based on students' personal perceptions of their experience and (b) there was not a control group. However this is in keeping with cooperative learning philosophy where the study design is predominantly of a qualitative nature.

## Results

### Group product evaluation summary

**Figure 1:**
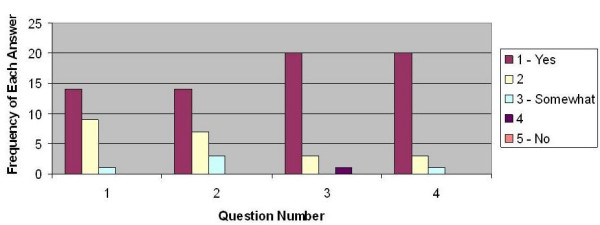
***Group Product Evaluation *Summary**. This is a graphic representation of the student group responses to the questions asked on the group product evaluation tool.

This survey was completed by 24 groups. Each group evaluated one poster (group product) other than their own. Students' perceptions about the group posters were that the information was presented clearly in easy understandable formats. Their comments were strongly favorable for the four criteria on the questionnaire. The results are graphically displayed in Figure 1.

• (Q1) Ninety-six percent of the posters (group product) were judged to have the information synthesized effectively.

• (Q2) Ninety-one percent of the posters were judged to have relevant information represented.

• (Q3) Ninety-six percent of the posters were judged to have the information is communicated clearly.

• (Q4) Ninety-six percent of the posters were judged to be easy to understand.

### Group process reflection summary

#### Quantitative assessment

**Figure 2:**
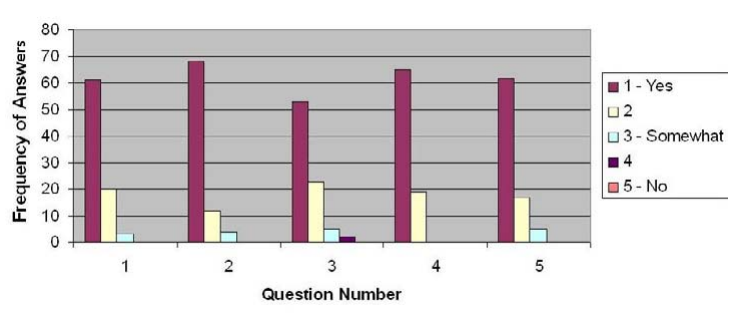
***Group Process Reflection *Summary**. This is a graphic representation of individual student responses to the questions asked on the group process reflection survey.

Individual students' perceptions about the functioning of their groups were strongly favorable (levels 1 and 2 on this survey) for the five criteria on the questionnaire. The results are graphically displayed in Figure 2.

(Q1) Ninety-six percent of the students (61 + 20 of 84 - level 1 + level 2) agreed that their team managed time effectively.

(Q2) Ninety-five percent of the students (68 + 12 of 84) agreed that they approached the task in a collaborative and cooperative way.

(Q3) Ninety percent of the students (53 + 23 of 84) agreed that they used appropriate and effective decision making strategies.

(Q4) One hundred percent (65 + 19 of 84) agreed that all team members contributed equally.

(Q5) Ninety-four percent of the students (65 + 19 of 84) agreed that the strategy they developed to approach the task was effective.

#### Qualitative Assessment

The comments offered by the students on the *Group process reflection *indicated a strong engagement and enthusiasm for the task in which they all participated willingly to the best of our knowledge. We categorized the comments in the following themes: group dynamics, learning, team management, and instructional design as listed in Appendix 3.

*a) Group dynamics*. Many students valued the opportunity to work with their peers in a non-threatening social atmosphere in which they had "fun." The students were surprised that they could have fun *and *learn.

*b) Learning*. Most of the comments indicated that learning was facilitated by this activity due to (i) a variety of unique perspectives, (ii) creating a visual product that solidified concepts and served as an examination study tool, and (iii) discussion. However, seven of the twenty-four comments in this category indicated that this activity was not a productive use of time. One individual felt "I would have learned more if I could have been more creative."

*c) Team management*. Students used a variety of strategies to facilitate decision-making processes and time management to maintain their group's integrity.

*d) Instructional design*. Student comments indicated that this task was a "good change." They suggested that the instructions be clearer and that there is less restraint on the requirements for how the poster was constructed.

### Midterm examination

Questions related to this content were analyzed in comparison to the remainder of the questions. Our underlying premise was that the use of CL in these content related questions would result in achieving at least a comparable correct answer response rate if not a better response rate than questions related to concepts where CL was not used as an instructional strategy.

Analysis was carried out with respect to the specifically targeted questions based on the material covered in the poster (9 out of 67). The correct response ranged from a low of 63 (70% of the students answered the question correctly) to a high of 83 (92% of the students answered the question correctly). This is in comparison of the correct response range of a low 10% to a high 100% on the questions based on the remainder of the examination. The two specifically targeted short-answer questions were correctly answered by 91% (82 correct responses) and 92% (83 correct responses) of the students respectively.

## Discussion

Cooperative learning is defined by the five key elements of positive interdependence, individual accountability, face-to-face interaction, group processing, and teaching inter-personal skills. There are objectives in both the task and group process domains. Small group teaching, which is widely employed in many phases of undergraduate medical education, is not synonymous with cooperative learning. Though small group teaching may address some of these elements by people working together as a team, it is often not purposefully designed to meet all the elements of cooperative learning. Yet, we believe that there is room for both team *and *cooperative approaches to group work in undergraduate medical education.

The majority of the students in this Pathology course (90–100%) agreed that their group process skills of time management, task collaboration, decision-making, and task execution were effective for this cooperative learning exercise. Likewise, the majority of the student groups (91–96%) judged the group products to be relevant, effective, easy to understand, and clearly communicated. Many of the students felt that the poster also served as a quick study review tool for the midterm examination.

In our study, most of the comments related to *learning *indicated that this task was overall a beneficial learning experience. This is similar to Gibson's findings [15] that professional net-working and group assisted learning which occurred in the hospital training for junior doctors was perceived to be a beneficial learning experience.

Seven of the twenty-four student comments in the *learning *category indicated that this activity was not a productive use of their time. These student perceptions could perhaps be attributed to:

a) non alignment of the given task with their personal preferred learning styles;

b) a threat or challenge to their traditional view of medical education; and

c) the students are predominantly high achievers who have succeeded quite well academically independently. Hernandez [17] reported that students resisted participating in team learning activities. This perhaps explains some of the negative or incongruent student remarks encountered in our study.

Sobral's study [16] found that preparing students to work in cooperative groups was a meaningful and productive use of time. The students involved in Sobral's study [16], however, had *chosen *this elective knowing they would work in cooperative groups which perhaps, better aligned their individual preferred learning styles (a priori). They were, thus, already "on board" with this approach and therefore may have felt that this contributed to their self-directed learning with enhanced group skills and team work. In our study, this task, which encouraged student ownership of learning, was part of a *compulsory *course which had been taught in the traditional lecture format up until now. The students in this Pathology course were slowly being introduced to more active forms of learning [6,18] and for this task, selected their own groups to minimize the potential for conflicts of personality and scheduling [19].

Some students indicated that the instructions could be clearer and there could be less restraint on the requirements for how the poster was constructed. One individual wrote that "I would have learned more if I could have been more creative." This may mean that the task was too restrictive for this student or that the group in which this student worked did not value this "creative look" despite in-group negotiation.

Resistance to shifting from the traditional faculty-driven curriculum for instructors with student ownership including shifting the learning environment from an individualistic competitive system to a cooperative non-competitive atmosphere is exemplified by the following anecdote. The day after the assignment was discussed in class, one student arrived at the instructor's office with the poster completed. The student was keen, enthusiastic, bright, and proud of his accomplishment. The instructor recognized and wanted to reinforce this enthusiasm but realized that the student had not in fact completed the required objectives for this assignment. The student was re-directed to work with his chosen group to create a *group *product. In a traditional competitive environment, the student would perhaps have been rewarded for his initiative [17].

We believe that cooperative learning is feasible and can be incorporated as an instructional strategy within the limited contact hours in the delivery of the content-laden undergraduate medical curriculum. As an instructional tool/activity, this has the potential to create a fun, revitalized, and dynamic learning environment for students and instructors alike. The faculty's role in cooperative learning is to (a) specify objectives, (b) decide on group size and how groups will be formed, (c) explain the task, (d) monitor students' learning, (e) encourage increased team work skills, (f) evaluate student learning, and (g) help students process how their groups functioned. Setting up this task takes careful, thoughtful planning and, therefore, requires dedicated faculty time to ensure success with this educational intervention. Ravenscroft *et al *[20] and Imel [21] noted teachers' reluctance to employ team learning methods in classes. Thus, we may also have to overcome, not only student resistance [17] but also may encounter a similar reluctance of faculty to use cooperative learning activities in medical education. This is further compounded by the paucity of evidence-based documentation and published articles related to such educational interventions in this discipline, which may impede instructors from embracing the value and in the utilization of such alternative strategies in undergraduate medical education.

The cycle of learning begins with the student being taught and ends with the student being assessed on what was taught. All educational interventions need to be assessed to ensure that despite varied modalities of instructional design the student is able to perform well on all standard assessment tools with the underlying principle being 'do no harm'. In this context, it was important for the instructor to evaluate the students' performance on the content of this section of the course at the standard required midterm examination in comparison to the remaining course content. It is for this reason that the measurement tools in this predominantly qualitative study included not only the evaluation of the group process and group product as per cooperative learning philosophy but also the standard quantitative assessment of student performance at the midterm examination. Thus, this study has mixed research design methodology to satisfy all concerns of the curriculum committee and the institutional review board. It was heartwarming to note that the course content handled by cooperative learning did not have any deleterious effects in students' performance at the standard midterm examination scores. Though a successful outcome on any given task is virtually guaranteed with highly-driven, high-achieving medical students, it cannot be assumed that this same population has the skills to participate effectively in group situations. This makes it all the more important to structure learning activities that offer opportunities to practice and develop interpersonal skills that are critical for effective team function.

## Conclusion

In conclusion therefore cooperative learning can be effective incorporated as a teaching strategy in the early years of undergraduate medical education. Co operative learning can address the desired outcomes in both content assimilation and development of interpersonal skills for medical students in their transition journey from being students to practicing physicians. This however, is a major shift from the traditionally held teaching and learning paradigms that is espoused in the medical education community. Such educational interventions may therefore pose challenges not only for students but faculty as well who have so far been socialized in a traditional reward system that acknowledges individual accomplishments in a competitive environment.

## Appendix 1

### Group Product Evaluation Survey Questionnaire

This is the form that students were given to evaluate the group product (the poster) using a modified Likert scale (see Additional File 1).

## Appendix 2

### Group Process Reflection Survey Questionnaire

This is the form that individual students were given to reflect on the group process. This form uses a modified Likert scale and invited written comments and feedback (see Additional File 2).

## Appendix 3

### *Group Process Reflection *Summary – Qualitative Analysis-Emergent Categories

The individual student responses to the group process reflection survey were listed and sorted according to main evolving themes of a) group dynamics, (b) learning, (c) team management, and (d) instructional design (see Additional File 3).

## Competing interests

The author(s) declare that they have no competing interests.

## Authors' contributions

Both authors have made substantial contributions to the conception/design of this study, acquisition, analysis and interpretation of data. Both authors have also have been involved in drafting the manuscript and revising it critically for intellectual content and have given final approval for publication of this version.

## Supplementary Material

Additional file 1 Group Product Evaluation Survey Questionnaire Click here for file

Additional file 2 Group Process Reflection Survey Questionnaire Click here for file

Additional file 3 *Group Process Reflection *Summary – Qualitative Analysis-Emergent Categories Click here for file
