# Supplementary material for: Cooperative learning in the first year of undergraduate medical education
Source: World J Surg Oncol. 2007 Nov 28;5:136. doi: 10.1186/1477-7819-5-136 (PMC2217551; doi:10.1186/1477-7819-5-136)
Supplement: Additional file 1 — Group Product Evaluation Survey Questionnaire [file 1477-7819-5-136-S1.pdf]

Appendix 1: Group Product Evaluation Survey Questionnaire
This is the form that students were given to evaluate the group product (the poster) using a modified Likert scale.

Q1 The information is synthesized effectively.

1---------------2--------------3 ----------------- 4 ---------------5
Yes Somewhat No

Q2All relevant information is represented.

1---------------2--------------3 ----------------- 4 ---------------5
Yes Somewhat No

Q3The presentation of the information is communicated clearly.

1---------------2--------------3 ----------------- 4 ---------------5
Yes Somewhat No

Q4The chart is easy to understand.

1---------------2--------------3 ----------------- 4 ---------------5
Yes Somewhat No
