# Supplementary material for: Cooperative learning in the first year of undergraduate medical education
Source: World J Surg Oncol. 2007 Nov 28;5:136. doi: 10.1186/1477-7819-5-136 (PMC2217551; doi:10.1186/1477-7819-5-136)
Supplement: Additional file 2 — Group Process Reflection Survey Questionnaire [file 1477-7819-5-136-S2.pdf]

Appendix 2
Group Process Reflection Survey Questionnaire
This is the form that individual students were given to reflect on the group process. This form uses a modified Likert scale and invited students' written comments and feedback.

Our team managed our time effectively.

1---------------2--------------3 ----------------- 4 ---------------5
Yes Somewhat No

We approached the task in a collaborative and cooperative way.

1---------------2--------------3 ----------------- 4 ---------------5
Yes Somewhat No

We used appropriate and effective decision making strategies.

1---------------2--------------3 ----------------- 4 ---------------5
Yes Somewhat No

All team members contributed equally.

1---------------2--------------3 ----------------- 4 ---------------5
Yes Somewhat No

The strategy we developed to approach the task was effective.

1---------------2--------------3 ----------------- 4 ---------------5
Yes Somewhat No

<u>Additional comments and observations:</u>

Positives:

Negatives:
