# Supplementary material for: Cooperative learning in the first year of undergraduate medical education
Source: World J Surg Oncol. 2007 Nov 28;5:136. doi: 10.1186/1477-7819-5-136 (PMC2217551; doi:10.1186/1477-7819-5-136)
Supplement: Additional file 3 — Group Process Reflection Summary – Qualitative Analysis-Emergent Categories [file 1477-7819-5-136-S3.pdf]

Appendix 3
***Group Process Reflection* Summary-- Qualitative Analysis-Emergent Categories**

The individual student responses to the group process reflection survey were listed and sorted according to main evolving themes of a) group dynamics, (b) learning, (c) team management, and (d) instructional design.

a) Group dynamics

Good opportunity to work as a team

Communicated well.

Got to know each other better.

Great time together.

Practice working as a group

Fun to do a group project.

I got to know my classmates better.

We had supper together…

We worked well together and enjoyed our time.

b) Learning

Very visual

Interesting to see how different groups approached/interpreted the same assignment.

Everyone contributed and gave valuable input.

Beneficial.

Different perspectives.

Everyone has unique ideas.

Different ideas for different posters.

Discussing the topic helped to solidify concepts.

Good to study from the poster.

Increased interest in learning for this arduous topic; fun to work together; less work than doing it by myself.

Team members helped explain things to each other.

Doing the summary is a more effective way to learn than reading the summary. (Since it was a group project, I only did one fourth of the summarization).

Material was straightforward with strict guidelines so it was hard to bring any creativity to the group.
The posters were very similar so we didn't learn much from each others posters.
No guidelines next time. The group that didn't follow the guidelines was the most interesting.
I would have learned more if I could have been more creative.
Difficult to do a computer project done in a group. Had it been done by hand it would have been far more effective.
I don't think this group project was a good use of our time (which is so limited for Pathology in the first place).
Didn't learn from other people—just learned from our own sections.
Could have used time better by studying alone.
Could have learned it better on my own.

c) Team management

Good way to manage time effectively and work together.
Met early on to establish a time frame of events and stuck to it.
Clear communication through email.
I wish we had more time in class to do it.
(Since it was a group project, I only did one fourth of the summarization).
Using a veto vote to make the decisions.
Some members of the group tried to control the final decisions but the group as a whole counteracted this to some degree.
Depended on one person to finish it up.
Final decisions made with group consensus.

d) Instructional Design

Good change.
Slight delay at the beginning.
Vague instructions.
The size was small for the project and complexity of the topic.
I think we wasted a lot of time in the hallway.
Good change from independent learning.
